# NLRP3 Inflammasome Signaling as a Link Between HIV-1 Infection and Atherosclerotic Cardiovascular Disease

**DOI:** 10.3389/fcvm.2020.00095

**Published:** 2020-06-11

**Authors:** Caroline Mullis, Talia H. Swartz

**Affiliations:** ^1^Department of Medicine, Icahn School of Medicine at Mount Sinai, New York, NY, United States; ^2^Division of Infectious Diseases, Department of Medicine, Immunology Institute, Icahn School of Medicine at Mount Sinai, New York, NY, United States

**Keywords:** inflammasome, HIV, NLRP3, atheroclerosis, inflammation

## Abstract

36.9 million people worldwide are living with HIV-1. The disease remains incurable and HIV-infected patients have increased risk of atherosclerosis. Inflammation is a key driver of atherosclerosis, but no targeted molecular therapies have been developed to reduce cardiovascular risk in people with HIV-1 (PWH). While the mechanism is unknown, there are several important inflammatory signaling events that are implicated in the development of chronic inflammation in PWH and in the inflammatory changes that lead to atherosclerosis. Here we describe the pro-inflammatory state of HIV-1 infection that leads to increased risk of cardiovascular disease, the role of the NLR Family Pyrin Domain Containing 3 (NLRP3) inflammasome in HIV-1 infection, the role of the NLRP3 inflammasome in cardiovascular disease (CVD), and outline a model whereby HIV-1 infection can lead to atherosclerotic disease through NLRP3 inflammasome activation. Our discussion highlights the literature supporting HIV-1 infection as a stimulator of the NLRP3 inflammasome as a driver of atherosclerosis.

## Introduction

Human Immunodeficiency Virus (HIV-1) causes a chronic infection that impacts more than 36 million individuals worldwide. HIV-1 treatment with highly active antiretroviral therapy (ART) can achieve virologic suppression leading to increased life expectancy in PWH. Still, PWH develop age-related co-morbidities including atherosclerosis and cardiovascular disease (CVD) earlier than their HIV-negative counterparts. This phenomenon is multifactorial including contributions from higher behavioral risk factors, ART toxicity, and chronic inflammation (1–6). Inflammatory signaling pathway activation has been described in the generation of chronic inflammation in PWH and in cardiovascular disease. The NLRP3 inflammasome pathway is activated in HIV-1 infection contributing to chronic inflammation and has also been implicated in atherosclerotic plaque formation. We present a potential role for NLRP3 inflammasome activation in chronic inflammation contributing to increased rates of CVD in PWH.

## HIV-1 Infection as a Pro-Inflammatory State

The early development of age-related co-morbidities in PWH can in part be attributable to the observed persistent inflammation and immune dysfunction seen in HIV-1 infection (1, 7, 8). HIV-1 infection creates a pro-inflammatory state marker by an increase in senescent cells that secrete inflammatory mediators resulting in low-level inflammation and increased T lymphocyte cell turnover (1, 9). Senescent cells secrete inflammatory mediators resulting in low-level inflammation and increased cell turnover (1, 10–12). Desai and Landay propose a multifaceted model for accelerated aging in HIV-1 infection (13): residual HIV-1 replication in activated immune cells despite ART; HIV-1 depletion of Th17 lymphocytes at GI epithelial mucosa causes microbial translocation and antigenic burden; thymic dysfunction causes loss of naïve T lymphocytes and regulatory CD4^+^ T lymphocytes, suppressing T lymphocyte activation; clonal expansion of activated immune cells with T lymphocyte loss of CD28 and telomere shortening, causing non-functional, senescent T lymphocytes (9, 14–17). Klatt et al. describe fibrosis and dysfunction of lymphoid organs and co-infection by pathogens such as cytomegalovirus (CMV) (4).

Both the viral reservoir and immune checkpoint molecule dysregulation mark an important mechanism for HIV-1 persistence and residual inflammation. HIV-1 results in productive infection of a small percentage of permissive cells with abortive infection of non-permissive bystander cells in lymphoid tissues (>95% CD4 T cell population). These bystander cells undergo pyroptosis, a programmed cell death, which could re-activate latently infected cells, causing more uninfected cells to die and sustain chronic inflammation (12, 18–24). Additionally an immune checkpoint molecule, programmed cell death protein 1 (PD-1) is highly expressed on CD4^+^ and CD8^+^ T lymphocytes during HIV-1 infection, and does not fully normalize on ART (12). On ART PD-1 levels correlate with CD4^+^ T lymphocyte count and are upregulated by Interleukin-7 (IL-7). Immune checkpoint molecules, lymphocyte-activation gene 3(LAG-3) and T cell immunoreceptor with Ig and ITIM domains (TIGIT), were also markers of HIV-1 infected CD4+ T cells on ART suggesting a role for immune checkpoint dysregulation in viral persistence.

## HIV-1 Infection and Cardiovascular Disease (CVD)

Cardiovascular disease is a major cause of morbidity and mortality in PWH (25–32). The D:A:D (Data Collection on Adverse Events of Anti-HIV Drugs) study estimates that 11% of deaths among PWH are attributable to CVD (33) and the Clinical and Virological Outcome of European Patients Infected With HIV (EuroSIDA) study estimates that one third of clinical events that are non-AIDS defining are related to cardiovascular disease (34). Risk of CVD in PWH is multifactorial with possible contributions from antiretroviral therapies (ART), increased exposures to traditional risk factors and chronic inflammation (35–39). In a meta-analysis, PWH when compared to HIV-1 uninfected control patients had an increased relative risk of CVD (40, 41). The D:A:D study also showed an increased relative risk of CVD with exposure the protease inhibitor drug class (33, 40) that mostly was driven by the increased CVD risk associated with ritonavir-boosted darunavir (42). The contribution of protease inhibitors to CVD risk has been controversial and limited by observational data. Nucleoside reverse transcriptase inhibitors (NRTIs) such as stavudine and zidovudine are associated with dyslipidemia, impaired insulin resistance, and greater carotid intima-media thickness (cIMT) in the setting of hyperlipidemia contributing to CVD risk (43–45). In the D:A:D trial abacavir was associated with increased risk of CVD; however, this association has not been observed in longitudinal data (40).

Higher rates of smoking, substance use and dyslipidemia contribute to increased risk of CVD in PWH (42, 46–48). PWH had 50% increased risk of acute myocardial infarction after controlling for behavioral and Framingham risk factors such as dyslipidemia, hypertension, and smoking suggesting additional mechanisms for increased CVD in PWH (49). However, some evidence suggests the association between smoking and CVD is even stronger in PWH (48).

Inflammatory biomarkers demonstrate chronic inflammation are associated with cardiovascular disease in PWH in large clinical cohorts (7, 35, 46, 50, 50–54). Compared to uninfected individuals PWH had 50% higher high sensitivity C-reactive protein (hsCRP), 150% higher Interleukin-6 (IL-6), 90% higher D-dimer, and 25% higher cystatin-C level. Inflammatory biomarkers like IL-6, hsCRP, and D-dimer remain elevated in PWH compared to uninfected controls despite ART treatment. D-dimer, hsCRP, and IL-6 have been associated with increased risk of CVD, and soluble CD14 (sCD14) has been associated with microbial translocation (7). In the Multicenter AIDS Cohort Study (MACS) of HIV+ men, IL-6, hsCRP, tumor necrosis factor alpha (TNF-α), soluble CD14 (sCD14), and soluble tumor necrosis factor receptor II (sTNFR II) were markers of frailty in HIV-1 disease, establishing an association with monocyte-macrophage immune activation in PWH (55). Treatment intensification has not resulted in improvement in HIV-1 viremia or inflammatory biomarkers (56, 57). Initiation of ART in patients who are elite controllers, those who spontaneously suppress HIV-1 viral load without ART, remains controversial (58–60).

The HIV-1 pro-inflammatory state outlined in the prior section also contributes to increased rates of CVD observed in PWH. The formation of atherosclerotic plaques contributing to CVD is an inflammatory process involving inflammatory signaling leading to monocyte recruitment, migration, and activation into pro-inflammatory foam cells (61, 62). HIV-1 proteins (*nef*, *tat*, and *env)* can induce inflammatory signals activating macrophages facilitating foam cell transformation and plaque formation (61).

In PWH, CVD has been associated with elevated inflammatory markers. In the Strategies for Management of ART (SMART) trial, a randomized, controlled trial assessing episodic ART therapy guided by CD4+ count compared to continuous ART, the observed hazard ratio of cardiovascular disease between the drug conservation group (episodic ART) against the viral suppression group (continuous ART) was 1.6 (*p* < 0.05) (50). This increase in CVD risk with episodic ART use was associated with an increase in inflammatory markers such as IL-6 (40, 63). Arterial inflammation is also modestly increased in PWH (59) and correlates with circulating inflammatory biomarkers such hsCRP and IL-6 but not with HIV-1 markers. In PWH initiated on ART, reduction of HIV-1 RNA correlated with decrease in D-dimer and IL-6 but not in hsCRP, while hsCRP was associated with progression of HIV-1 and mortality after adjustment for CD4^+^ count and HIV-1 viral load (64). These studies suggest a role for HIV-1 related inflammation as a contributor to CVD risk in PWH.

Decreasing inflammation represents an important therapeutic strategy for CVD prevention (26, 65). In the JUPITER trial patients with low LDL and high hsCRP were randomized to statin vs. placebo. Statin treatment had 44% relative risk reduction (66, 67). In the SATURN-HIV trial of rosuvastain in PWH, cIMT reduction on statin compared to placebo was independent of the lipid lowering effects (66, 68). With long-term statin use (48 weeks) almost all inflammatory markers decreased, including sCD14. By contrast aspirin did not decrease markers of inflammation including sCD14, IL-1 and D-dimer in a smaller trial of 12 week follow-up (69). Table 1 summarizes therpies that have been tested to reduce inflammation as a cause of atherosclerosis in HIV-1 disease.

**Table 1:** Drugs with anti-inflammatory properties to reduce atherosclerosis in HIV disease.

**Mechanism of action**	**Drug examples**	**References**	**Notes**
**COAGULATION**			
COX-1 pathway inhibition	Aspirin	(69, 92)	No differences in soluble markers (sCD14, IL-6, sCD163, D-dimer) or T-cell or monocyte activation.
Adenosine reuptake inhibitor	Dipyramidole	(93)	Decreased CD4^+^ T-cell activation in pooled analysis. No changes in soluble markers (sCD14, IL-6, sCD163, CRP, IL-10, sCD27, D-dimer)
Factor Xa inhibitor	Edoxaban	(94)	No differences in inflammation (IL-6, TNF-RI, IL-1β, sCD163, sCD14, or monocyte activation markers. Lowered D-dimer and thrombin antithrombin (TAT).
Coagulation inhibition Factor IIa inhibitor	Dabigatran	(95, 96)	Attenuated atherosclerotic plaque formation, decreased collagen content and ROS production, observed improved endothelial function
**METABOLISM**			
Inhibits dihydrofolate reductase enzyme Inhibits binding of IL1β to its surface receptor	Methotrexate	(97, 98)	No significant effect on endothelial function or inflammatory biomarkers (hs-CRP, IL-6, IP-10, sCD163, sCD14, D-dimer, fibrinogen, VCAM) associated with decreased CD8+ T-cells, saw more safety events (Hsue) LDMTX with some effect on brachial artery US that correlated with decreased D-dimer
HMGCoA enzyme inhibition	Statins	(99, 100)	Decreased sCD14 and IP-10 levels, decreased activated T-cells (Funderberg); Reduction in non-calcified plaque volume and high-risk coronary plaques (Lo); Reducing ASCVD risk, ongoing REPRIEVE trial
Inhibition of ATP-citrate lyase and activation of AMP activated protein kinase in the liver	Bempedoic acid	(101–103)	Prevention of atherosclerotic plaque development and associated inflammation; lowers LDL, total cholesterol, apolipoprotein B, hs-CRP- unclear clinical effect
**CYTOKINE SIGNALING**			
mAB blocking IL-1β	Canakinumab	(104, 105)	Lower rates of recurrent CVD independent of lipid lowering, higher incidence of fatal infection, expensive therapy Decreased rates of hs-CRP, IL-6 and sCD163, no impact on T cell activation or monocyte subsets Decreased arterial inflammation on FDG-PET
mAB binding IL-6	Tocilizumab	(106)	Expensive therapy, effective for treatment of Castleman disease; reduced levels of secretory phospholipase A2-IIA, lipoprotein (a), fibrinogen, D-dimers, elevated paraoxonase; increased LDL and triglyceride levels
Jak-inhibitors	Ruxolitinib/tofacitinib/baricitinib	(107)	Ruxolitinib with no decrease IL-6 levels, decrease in sCD14, increase in circulating T- cells
IL-1R	Anakinra	(108)	Improved myocardial deformation; decreased hs-CRP at time of NSTE-ACS
TNF-alpha inhibitors	Infliximab—Etanercept—Adalimumab	(109)	Increased total cholesterol and HDL levels in RA patients; no change in CRP levels, potentiated response to acetylcholine
**COINFECTIONS**			
Competitive inhibitor of deoxyguanosine triphosphate inhibiting viral DNA polymerases	Valgancyclovir	(110)	Reduced CD8 activation, no significant difference in CRP
**GUT MICROBIOME**			
Alteration of microbiome	Probiotics	(111, 112)	Increase in Th17 cell subsets; Lipopolyscharide binding protein and hs-CRP decrease with probiotics in PWH, not sCD14 and D-dimer; Increase in serum serotonin, decreased tryptophan in plasma, reduction in CD38 and HLA-DR expression on PBMCs
Antibiotic	rifaximin	(113)	No effect on LPS (lipopolysaccharide) and sCD14 at 2 weeks, decrease LPS in cirrhotic patients

## HIV-1 and the NLRP3 Inflammasome

Activity of the NLRP3 inflammasome contributes to the chronic, pro-inflammatory state in PWH (70–75). The inflammasome is part of the disease through the innate immune system activated by pattern recognition receptors (76). The inflammasome activates caspase-1, which cleaves prointerleukin-1β (pro-IL1β) into the mature, secretory interleukin-1β (IL-1β). Inflammasome activation also mediates pyroptosis or programmed cell death of myeloid and lymphoid cells.

HIV-1 infection provides the first of two signals for NLRP3 inflammasome activation (77). Monocyte-derived macrophages primed with HIV-1 have increased IL-1β production after exposure to the second NLRP3 activation signal. HIV-1 virions induce Toll-Like Receptors (TLRs) to stimulate pro-IL-1β expression (71, 78). HIV-1 infection is required for activation as, when exposed to ART, induction of pro-IL-1β and release of IL-1β were decreased (78). HIV-1 and HCV virion induction of TLRs may not be dependent on cell entry as induction of the inflammasome was still seen in the presence of cell entry inhibitors (71).

The NLRP3 inflammasome has been well-studied as a cause of T lymphocyte cell death and activation through pyroptosis (19, 21, 22, 24, 79, 80). This has been demonstrated as infected CD4 T lymphocytes can stimulate bystander cell NLRP3 inflammasome activation and stimulation of pyroptosis (19, 21, 22, 24). The role of the NLRP3 inflammasome has been studied in HIV pathogenesis in lymphoid tissue and in peripheral blood (81). Peripheral blood monocytes in PWH were positive for an inflammasome adaptor protein, ASC speck (apoptosis-associated speck-like protein containing a caspase-recruitment domain), not seen in healthy controls (82). ASC speck protein is a marker for inflammasome activation, suggesting that in PWH activation of pyroptotic cell death is responsible for progressive CD4^+^ T lymphocyte death and contributes to chronic inflammation.

Inflammasome activation occurs during acute HIV-1 infection (83) and persists in immune non-responders, patients on ART without CD4^+^ T lymphocyte recovery (CD4 < 350) (74). When stimulated with lipopolysaccharide (LPS), a TLR signal for inflammasome activation, upregulation of inflammasome genes (NLRP3, caspase-1) was seen in both immune non-responders and responders (CD4 > 500). Substance abuse enhances inflammasome activation in PWH. Cocaine exposure to HIV-1 infected macrophages increases activity by potentially priming the NLRP3 inflammasome by potentiating reactive oxygen species (ROS) production (84, 85). By contrast, cannabis has been demonstrated to reduce NLRP3 inflammasome activation (86, 87).

## CVD and the NLRP3 Inflammasome

Several studies implicate the NLRP3 inflammasome in the pathogenesis of atherosclerosis (88). The role of the NLRP3 inflammasome in atherosclerosis was demonstrated using low-density lipoprotein (LDL) receptor deficient mice, a model for familial hypercholesterolemia (89). After lethally irradiated bone marrow was reconstituted with wild-type, NLRP3-, ASC-, or IL1α/β-deficient bone marrow, mice had lower levels of IL-18 and IL-1 family cytokines and showed decreased atherosclerosis. IL-1β release was observed after 24-h incubation with LDL even in the absence of other known NLRP3 inflammasome primers suggesting a role for cholesterol in both priming and activation of the inflammasome pathway.

Interestingly, in an *in vivo* model *ApoE*^−/−^*, Nlrp3*^−/−^, *ApoE*^−/−^*, Asc*^−/−^*, ApoE*^−/−^, and *caspase-1*^−/−^ double-deficient mice fed a high-fat diet failed to demonstrate differences in atherosclerosis progression and phenotype of the plaque (90). The differences in these findings could be attributed to the use of different mouse models and a potential role of IL-1α, which can be generated independently of the NLRP3 inflammasome. These contradictory results have also been attributed to different diets as in the Menu et al. study mice were fed 1.25% cholesterol (HFD) diet which could have overwhelmed genetic differences as compared to the 0.15% cholesterol diet (western diet) (91).

Inflammasome activity in plaque generation is further supported by the presence of activated caspase-1 in atheromatous plaques and caspase knockout models in *ApoE*^−/−^ mice (114–116). Caspase knockout in *ApoE*^−/−^ mice decreased the size of atherosclerotic lesion size in the aortic arch, intra-lesion IFN-γ and plasma levels of IL-1β and IL-1α. In *ASC*^−/−^ mice, the NLRP3 inflammasome contributes to atherogenesis by triggering maturity of IL-1β and IL-18 in atherosclerotic plaques after vascular injury (117, 118). In *ASC*^−/−^ mice neointimal formation was attenuated and decreased IL-1β and IL-18 expression was observed in the plaques compared to wild type. Inhibition of NLRP3 inflammasome with arglabin, a plant-based metabolite inhibitor of the NLRP3 inflammasome, showed decreased atherosclerotic lesions in apolipoE2-Ki mice fed a high fat diet (118, 119).

Other activating factors have been identified. Trimethylamine-N-oxide (TMAO), a by-product of choline and L-carnitine metabolism, promotes the formation of foam cells from macrophages in a process mediated by inflammasome activity (120). TMAO stimulated thioredoxin-interactive protein (TXNIP)-NLRP3 inflammasome activity in human umbilical vein endothelial cells causing release of IL-1β and IL-18 in a dose and time dependent manner. LPS-exposed THP-1 macrophages induced Lectin-like oxLDL receptor-1 (LOX-1) expression, generation of ROS, auto-phagosome formation and damage to mitochondrial DNA (121). LOX-1 inhibition resulted in attenuated NLRP3 inflammasome activity consistent with decreased atherosclerotic plaque burden in LOX-1 deletion in mice fed a high fat diet.

Oxidative stress-responsive transcription factor NF-E2 related 2 (*Nrf2*) also has a role in inflammasome activation and atherosclerosis (122, 123). *Nrf2-/ApoE-* mice showed attenuation of atherosclerosis without change in lipid metabolism or foam cell transformation when compared to *Nrf2*^+^*/ApoE*^−^ mice. Cholesterol crystals triggered production of IL-1α and IL-1β in *Nrf2*^+/+^ dendritic cells, not observed in *Nrf2* deficient dendritic cells. In NLRP3-deficient and caspase1^−/−^ macrophages, cholesterol crystal-induced IL-1β production was reduced. Neutralization of IL-1α and IL-1β by induction of neutralizing antibodies resulted in reduced atherosclerosis in *Nrf2*^+/+^ *ApoE*^−/−^ but not in Nrf2^−/−^ *ApoE* ^−/−^ mice. These results suggest that the observed *Nrf2* effects on atherogenesis are from its role in inflammasome activation and IL-1 production (122).

Atherosclerosis is also associated with endothelial dysfunction. Endothelial senescence, which is linked to CV diseases, is associated with NLRP3 activation (124). Induction of endothelial cell senescence with bleomycin showed increased IL-1β and caspase-1. IL-1β promoted endothelial cell senescence as indicated by upregulation of p53/p21 expression.

Inflammasome activity in CVD is also modulated by separate cardiovascular risk factors including hyperglycemia, obesity and hyperuricemia (125). IL-1β is elevated in patients with high blood pressure and type 2 diabetes mellitus (118, 126). In type 2 diabetes use of γ-tocotrienol to inhibit the NLRP3 inflammasome can delay disease progression (127). Diabetic rats showed significantly increased NLRP3 inflammasome activation (118, 128). When animals were given rosuvastatin both NLRP3 inflammasome and MAPK expression was decreased, with associated decrease in cardiac fibrosis, suggesting a potential role for the NLRP3 inflammasome in diabetic cardiomyopathy.

There is literature supporting the role of the NLRP3 in hypertension. Attenuation of NLRP3 and caspase by chronic inhibition of NF-kB attenuates high salt induced hypertension (129). In a murine model of hypertension, a highly selective SGK1 inhibitor, EMD638683, was shown to suppress IL-1β release, NLRP3 expression, and caspase-1 activation which was associated by reduced transformation of fibroblasts to myofibroblasts (130). These effects on cardiac fibrosis were not observed with supplementation of exogenous IL-1β suggesting NLRP3 and IL-1β have a role in hypertensive cardiac damage.

The NLRP3 inflammasome and the associated inflammatory response have a role in the pathophysiology of a myocardial infarction (MI) (131, 132). During MI, release of cellular debris and production of reactive oxygen species activate the NLRP3 inflammasome leading to development of cardiomyopathy. Sandanger et al. demonstrate the role of the inflammasome in a murine myocardial ischemia-reperfusion injury, demonstrating NLRP3, IL-1β, and IL-18 mRNA expression was increased in cardiac fibroblasts post-MI (131). Cardiac fibroblasts had dose-dependent mRNA expression of NLRP3 and IL-1β when incubated with TLR2 and TLR4 ligands; this was blocked when incubated with NF-kB inhibitor. Post-MI, *NLRP3*^−/−^ mice demonstrated improved cardiac function and reduced infarct size compared with wild type after reperfusion. NLRP3 inflammasome activation may play a cardioprotective role in ischemia-reperfusion injury (133). IL-1β neutralizing antibodies and anakinra, an IL-1 receptor antagonist, showed reduced cardiac hypertrophy and myocardial dysfunction post-MI; this suggests potential therapeutic interventions to ameliorate cardiac dysfunction post-MI (118, 134, 135).

Therapeutic interventions targeting reduction of inflammation to reduce CVD risk have been tested. The JUPITER trial was a primary-prevention trial randomizing patients with a low LDL cholesterol (<130 mg/dL) but elevated hsCRP (>2.0 mg/L) to receive rosuvastatin or placebo (67). Rosuvastatin was associated with reduction in cardiovascular risk (hazard ratio 0.56), lower LDL, and hsCRP. Simvastatin reduced NLRP3 activation in a diabetic rat model (136). In addition to lowering LDL, statins may decrease CVD by decreasing inflammasome activity.

The NLRP3 inflammasome is also a potential therapeutic target. NLRP3 inflammasome inhibitors show potential in murine models. Arglabin, showed decreased IL-1β plasma levels and decreased atherosclerotic lesion size in an ApoE knockout (119). MCC950, a selective inhibitor of NLRP3, demonstrated decreased plasma IL-1β levels and decreased atherosclerotic plaque size and volume (137). The CANTOS trial randomized patients with past MI and elevated hsCRP on optimal lipid lowering therapy to receive canakinumab, a monoclonal antibody targeting IL-1β, or placebo (104). A 15% reduction in major CV events was observed without change in LDL cholesterol. The benefit of this therapy associated with higher risk of fatal infection. Subsequently numerous reports have implicated a role of targeting IL-1β in reducing atherosclerotic disease (104, 138–148). Most recently, Li et al. demonstrated that treatment with VX-765, an NLRP3 inflammasome inhibitor, halted progression of atherosclerosis and reduced vascular smooth muscle cells (VSMCs) pyroptosis (149).

## HIV, NLRP3 Inflammasome, and CVD

An emerging literature supports the role of the NLRP3 inflammasome as a driver of CVD in HIV-1 disease (150, 151). Yearley et al. demonstrated that in SIV-infected rhesus macaques, IL-18 secretion as a marker of NLRP3 inflammasome activity was associated with atherosclerotic progression (152). Further, they observed that the IL-18 was observed to be associated with macrophages, not T lymphocytes, suggesting the activation of alternative cell types in response to HIV-1 infection of T lymphocytes. Kearns et al. demonstrated that expression of HIV-1 transcripts can drive atherosclerosis through activation of caspase-1 in inflammatory monocytes. They further demonstrated that IL-18 levels were higher in HIV-1 infected patients with atherosclerotic disease and that these levels were correlated with monocyte/macrophage activation markers.

In a follow up study to the CANTOS trial, Hsue et al. demonstrated that a single dose of canakinumab reduced numerous inflammatory markers including cytokine production and atrial inflammation in PWH (105). This study and others suggest that inflammasome targeted therapeutic interventions reduction of cardiovascular events demonstrate a crucial role for this pathway in the pathogenesis of CVD in PWH. Hoel et al. evaluated PWH and demonstrated that soluble markers of interleukin 1 (IL-1Ra) levels were associated with a 1.5-fold increased risk of first-time myocardial infarction (153).

## Conclusions

**Figure 1:**
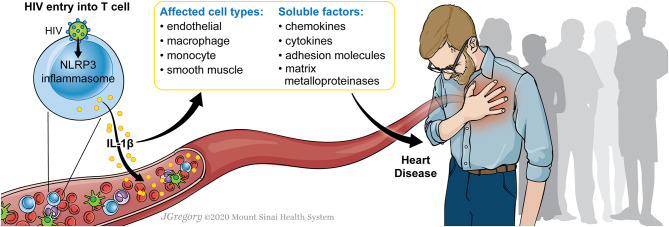
Model for HIV-1 infection and NLRP3 inflammasome activation to drive atherosclerosis. A model is shown in which HIV-1 infection of a CD4+ T cell can stimulate the NLRP3 inflammasome. This results in IL-1β secretion which impacts on various cell types including endothelial cells, macrophages, monocytes, and smooth muscle, signaling through soluble factors including chemokines, cytokines, adhesion molecules, matrix metalloproteinases. The activation of these factors can potentiate progression of atherosclerotic disease.

Here we describe the evidence supporting HIV-1 infection as a chronic and pro-inflammatory state that is associated with increased risk of atherosclerosis. We review the evidence defining the role of HIV-1 infection in activation of the NLRP3 inflammasome and the role of NLRP3 activation in the development of atherosclerosis. HIV-1 infection can mediate NLRP3 inflammasome activation, thus potentiating a pro-inflammatory state and increasing risk of atherosclerosis. Figure 1 demonstrates a model whereby HIV-1 infection can stimulate the NLRP3 inflammasome to result in IL-1β secretion which impacts on various cell types including endothelial cells, macrophages, monocytes, and smooth muscle, signaling through soluble factors including chemokines, cytokines, adhesion molecules, matrix metalloproteinases to result in progression of atherosclerotic disease. HIV-1 chronic inflammation, in concert with other factors, can drive inflammation including that seen in cardiovascular disease and that targeting this pathway can have important therapeutic benefits.

We present the data implicating the NLRP3 inflammasome as a signaling pathway that is both activated by HIV-1 infection and that drives the development of atherosclerosis in PWH. Gaps exist in our understanding to establish clear links between HIV-1 infection, NLRP3 inflammasome activation, and atherosclerotic disease with the approach of developing targeting therapies to reduce the inflammatory signaling that drives this important comorbidity. Animals studies of humanized mice that connect the risk of HIV-1 infection, NLRP3 activation, and the development of atherosclerosis are necessary.

The above studies highlight the importance of cardiovascular risk in PWH and the need for mechanistic understanding behind targeted therapies. Care of PWH should continue to focus on approaches to reduce cardiovascular risk in PWH through lifestyle modifications, tight lipid, hypertension, and glycemic control, while seeking to further identify biomarkers with linked clinical outcomes.

## Author Contributions

CM and TS conceived the idea and wrote the manuscript.

## Conflict of Interest

The authors declare that the research was conducted in the absence of any commercial or financial relationships that could be construed as a potential conflict of interest.

## Abbreviations

PWH: people with HIV-1
hsCRP: high sensitivity C-reactive protein
CVD: cardiovascular disease
ART: Antiretroviral therapy
NLRP3: NLR Family Pyrin Domain Containing 3.
